# Etiologic Causes and Epidemiological Characteristics of Patients with Intraocular Foreign Bodies: Retrospective Analysis of 1340 Cases over Ten Years

**DOI:** 10.1155/2018/6309638

**Published:** 2018-01-31

**Authors:** Lin Li, Hai Lu, Kai Ma, Yun-Yun Li, Hai-Yan Wang, Ning-Pu Liu

**Affiliations:** Beijing Tongren Eye Center, Beijing Tongren Hospital, Beijing Ophthalmology and Visual Sciences Key Laboratory, Capital Medical University, Beijing 100730, China

## Abstract

**Objective:**

Patients with intraocular foreign bodies were retrospectively analyzed. Population characteristics, pathogenic factors, and the outcomes during the past ten years were discussed.

**Design:**

Retrospective case series study.

**Method:**

Medical records of 1340 patients hospitalized in Beijing Tongren Hospital from January 1, 2004, to December 31, 2013, were collected.

**Results:**

Average age was 33.0 ± 13.8 (1–76) years old in 1340 patients. There were more males (1270, 94.8%) than females (70, 5.2%). Patients from outside of Beijing (82.1%, *n* = 1100) prevailed. Farmers (32.1%, *n* = 430) and workers (22.3%, *n* = 299) were the top two affected professions. Leading two causes were splashing of foreign bodies (SFB) (58.6%, *n* = 785) and explosives (31.8%, *n* = 426). More males than females were injured by SFB (59.4% versus 44.3%, *P* = 0.009). Firework injury was the commonest (41.6% versus 3.1%–15.3%, *P* < 0.05) in patients under nine. The annual percentages in patients over 50 increased (*P* < 0.001) and in patients by explosives decreased (*P* = 0.027).

**Conclusion:**

Most patients in this study were young males from outside of Beijing and farmers. SFB accounted most for patients over 10 years old and fireworks for those under ten. Patients over 50 increased while those by explosives decreased annually over the period.

## 1. Introduction

Intraocular foreign bodies (IOFB) cause a serious ocular trauma that can lead to blindness [1] and account for 10–40% of all open eye injuries [1–6]. IOFB is closely related to the living and working environment, as well as the individual awareness of protection and protective measures [7, 8]. However, there is little existing literature that analyzes patients with IOFB and the causes. For a better understanding of the population characteristics and causes of IOFB, we collected the data of hospitalized IOFB patients in Beijing Tongren Hospital from 2004 to 2013 for retrospective analysis. We also analyzed the trending changes of these characteristics in the hope to gain better insight into IOFB so as to generate theoretical bases for formulating protective measures against IOFB.

## 2. Subjects and Methods

Medical records were collected from all patients diagnosed with ocular trauma and hospitalized in Beijing Tongren Hospital from January 1, 2004, to December 31, 2013. Among them only patients with IOFB were retrospectively analyzed. The diagnosis of IOFB was defined based on Birmingham Eye Trauma Terminology (BETT) [9]. IOFB included the foreign bodies in the anterior segment (anterior chamber, iris, and lens) and the posterior segment (vitreous body, retina, and subretina). Direct diagnosis of IOFB depended on the discovery of foreign bodies through a slit lamp microscope, direct or indirect ophthalmoscope, or operation microscope. Indirect diagnosis of IOFB was based on the medical history and ophthalmologic imagery [10, 11], such as computed tomography (CT) scan, eye ultrasound, ultrasound biomicroscopy (UBM), and X-ray of the eye. CT has high spatial resolution and low contrast resolution. Most of the IOFB (such as metal or glass debris) can be diagnosed and located accurately by transverse, coronal CT images and sagittal images of thin-layer recombination. According to the position and characteristics of IOFB, different surgical methods were selected, such as vitrectomy or combined with cataract extraction, the anterior chamber foreign body removal or combined with cataract extraction, and magnetic suction through scleral incision. Best-corrected visual acuity (BCVA) of preoperation and 1 week postoperation was recorded. A database of patients with IOFB was established using EpiData Entry 3.1 with double entry. Items included registration number, personal information, ophthalmic examinations, history of diseases, and so forth. The Declaration of Helsinki was followed in this research with all medical records anonymized and all the information designated for research purposes only.

Patients were grouped by a number of traits. By age, we divided patients into seven groups: 0–9, 10–19, 20–29, 30–39, 40–49, 50–59, and over 60. We also grouped patients by their self-reported professions as workers, farmers, freelancers, employees, students, preschool children, and other professionals (i.e., professions with few cases of IOFB and therefore inconvenient for classification). By causes of IOFB, we classified patients into four categories: splashing of foreign bodies, explosive, stabbing, and others. Among them, explosive was further divided into that of fireworks and other explosives, which included all explosives other than that of fireworks such as those of detonators, gunpowder, tires, and glasses; others referred to causes that led to few patients and those still unclear. Patient residences were documented according to patient-provided information.

## 3. Statistical Analysis

SPSS 22.0 (SPSS Inc., Chicago, IL, USA) was used for statistical analysis. The EpiData database was imported. All data underwent descriptive statistics, parameter comparison, and nonparametric comparison. Chi-square test was used for analyzing rates, while parametric variables were evaluated by one-way ANOVA. *Z*-test was employed for analyzing causes for the whole sample, by profession and by age group. Trend analysis was made using chi-square trend test. All *P* values were two tailed with values less than 0.05 considered as statistically significant.

## 4. Results

**Figure 1:**
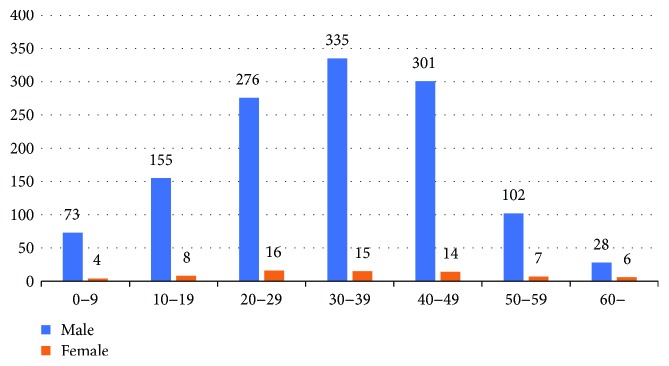
Age group distribution.

**Figure 2:**
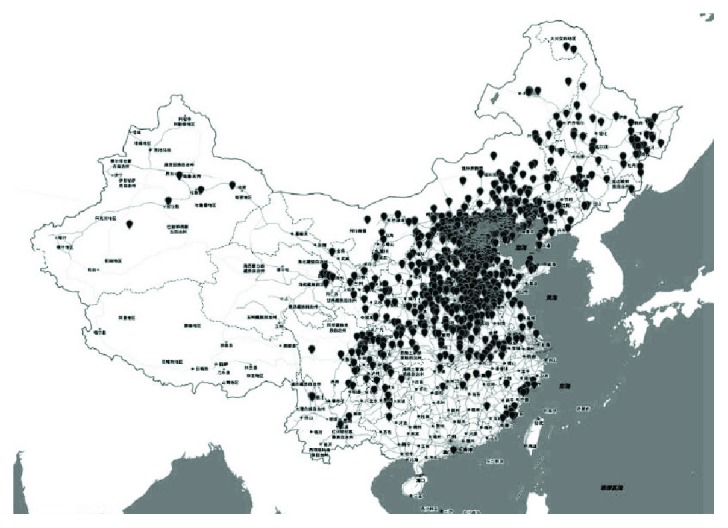
Distribution of patient residences.

Altogether there were 15,017 ocular trauma patients, 5917 open eye trauma, hospitalized in Beijing Tongren Hospital from January 1, 2004, to December 31, 2013. Among them, 1340 patients were diagnosed as having IOFB which was 22.6% in open eye trauma. The male/female ratio was 18.1 : 1 with 1270 (94.8%) males and 70 females (5.2%) with IOBF. Patient age ranged from 1 to 76 years with an average of 33.0 ± 13.8. For age group distribution, there were most patients aged 30–39 (6.1%, *n* = 350), followed by those aged 40–49 (23.5%, *n* = 315) and 20–29 (21.8%, *n* = 292); the total of which was 957 (71.4%) (Figure 1). Apart from an international patient from Mongolia, patients were from 27 of the 34 administrative regions in China except Shanghai, Hunan, Jiangxi, Tibet, Hong Kong, and so forth. Altogether, there were 240 (17.9%) Beijing residents and 1100 (82.1%) patients from outside Beijing. There was a radial distribution pattern with Beijing as the focus. As the distance between Beijing and a place increased, the number of patients from there decreased gradually. Most patients lived to the north of the Yangtze River (Figure 2).

**Figure 3:**
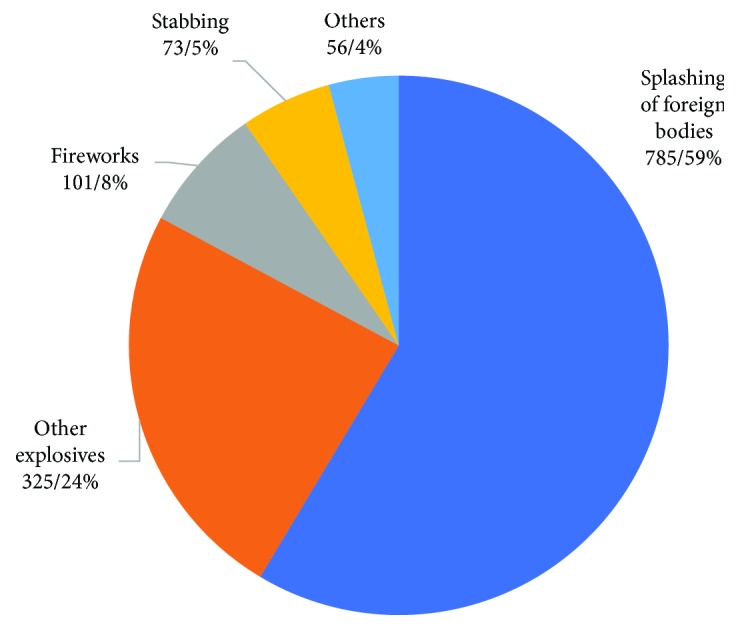
Distribution of causes.

Top two professions in patients with IOFB were farmers (32.1%, *n* = 430) and workers (22.3%, *n* = 299). Splashing of foreign bodies was the leading cause of injuries (58.6%, *n* = 785), followed by explosives (31.8%, *n* = 426). Among patients injured by explosives, there were 101 (7.5%) by fireworks and 325 (24.3%) by other explosives (Figure 3).

In the group of patients with IOFB, 644 cases (48.1%) were magnetic foreign bodies, 584 cases (43.6%) were nonmagnetic foreign bodies, and 112 cases (8.4%) were magnetic uncertain. Nonmagnetic foreign bodies included explosives (20.4%, *n* = 273), metal (5.8%, *n* = 78), glass (4.4%, *n* = 59), eyelashes (4.3%, *n* = 57), gravel (3.9%, *n* = 52), wood chips (1.2%, *n* = 16), other (1%, *n* = 14), cinder (0.7%, *n* = 10), plastic (0.7%, *n* = 10), and uncertain properties (1.1%, *n* = 15).

The percentage of male patients injured by splashing of foreign bodies was higher than that of female patients (59.4% versus 44.3%, *P* = 0.009), while a higher percentage of female patients were victims of stabbing (14.3% versus 5.0%, *P* = 0.001) (Table 1). For patients of 0–9 years old, injuries from fireworks were the most common (41.6%, *n* = 32/77), which were significantly more frequent than patients in other age groups (3.1%–15.3%, *P* < 0.05), while patients aged 10 or older were more often injured by splashing of foreign bodies (49.1%–70.6%) than those under 10 (16.9%, *P* < 0.05) (Table 2). Fireworks were the leading cause of injury among preschool and student patients (35.7%, *n* = 10/28; 29.9%, *n* = 47/157), as in significant contrast with other professions (2.0%–6.7%, *P* < 0.05). However, the percentages of preschool and student patients injured by splashing of foreign bodies (25%, 7/28; 24.2%, 38/157) were significantly lower than those of other professions (61.5%–69.4%, *P* < 0.05). Moreover, a significant smaller percentage of preschool patients (3.6%, 1/28) than that of other professions (15.1%–29.4%, *P* < 0.05) were injured by other explosives (Table 3).

**Figure 4:**
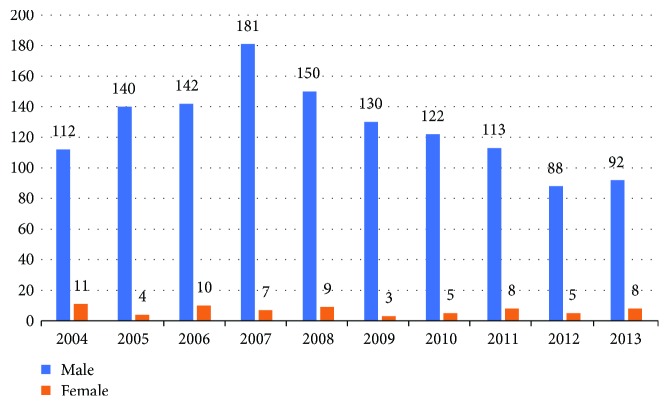
Annual number of patients by gender.

Over the ten years, male patients accounted for 91.1%–97.7% annually with no statistically significant variation (Figure 4). For age group distribution over the years, the annual percentage of patients aged 10–19 was decreasing (*P* < 0.001) while that of patients aged 50–59 and over 60 was increasing (*P* < 0.001), and no statistically significant variation was found in the annual percentages of patients in other age groups (Table 4). As for profession distribution over the years, there was a decreasing trend in the annual percentages of worker and student patients (*P* < 0.001; *P* = 0.027) and a climbing tendency in those of employee and freelancer patients (*P* < 0.001) (Table 5). In terms of causes, the percentages of patients injured by explosives as a whole (*P* = 0.027) and by other explosives (excluding fireworks) (*P* = 0.005) were declining over the years while those of patients injured by other causes did not vary in a statistically significant way (Table 6).

Endophthalmitis occurred in 184 cases (13.7%). Retinal detachment occurred in 486 cases (36.3%) in the group. Foreign bodies located in the posterior segment occurred in 1194 cases (84%), and foreign bodies located in the anterior segment occurred in 146 cases (10.9%). 1165 cases (86.9%) underwent vitrectomy or combined with cataract extraction, 140 cases (10.4%) were removed foreign bodies through anterior chamber or combined with cataract extraction, 9 cases (0.7%) underwent magnetic suction through scleral incision, 12 cases (0.9%) underwent eye enucleation, and 14 cases (1%) without surgery. In 1235 cases (92.2%) in the group, foreign bodies were taken out successfully, while in 91 cases (6.8%), foreign body was not removed. There were no significant differences in patients with preoperative and postoperative visual acuity less than 0.02 (69.9% versus 66.5%, *P* = 0.06).

Postoperative blindness (BCVA < 0.02) in 0–9-year-old patients was lesser in patients above 10 years old (50.6% versus 67.5%, *P* < 0.01). Patients with foreign bodies in the anterior segment have lower postoperative blindness than that of the patients with foreign bodies in the posterior segment (35.6% versus 70.3%. *P* < 0.01). Postoperative blindness in patients with blast injury was higher than the other causes of injury (75.4% versus 62.4%, *P* < 0.01). Postoperative blind proportion in patients with endophthalmitis was higher than that of patients without endophthalmitis (84.2% versus 63.7%, *P* < 0.01). Postoperative blindness proportion in patients with retinal detachment patients was higher than that of the patients without retinal detachment (88.3% versus 54.1%, *P* < 0.01).

## 5. Discussion

As a developing country, China has a large population of professionals in industry and agriculture, which play important roles in its national economy. For lack of protective measures at work, there is a high incidence of IOFB in these people. In this study, the proportion of IOFB in open ocular trauma is close to that reported in the literature [1–6]. Since there is no unified eye trauma registry system in China, it is impossible to obtain the accurate characteristics of IOFB patient population and the causes. Although this research only included medical records of hospitalized IOFB patients in Beijing Tongren Hospital, judged from the distribution of patient residences, the characteristics generated is meaningful not only for the patient population in Beijing but also for that to the north of the Yangtze River.

Males at working ages, especially young males, accounted for most of the patient sample, which was consistent with previous reports. The percentage of male patients (94.8%) was consistent with previous reports (90%–100%) [2, 4, 12–14]. This is probably because compared with females, males engage more in physical work and a higher risk of eye trauma ensues [15]. In patients over 60 years old, although the number of male patients was still higher than that of females, the percentage of males was slightly lower than that in other age groups. Tielsch et al. also reported a similar result but offered no clear explanation [16]. For this research, it is likely because males usually retire at 60 in China and engage less in physical work after retirement. The average and peak ages of IOFB patients in this research are consistent with previous reports (29–42 [2, 4, 5, 12, 17]; 30–50 [18–21]). This is probably because people of these ages engage more in physical work. In regard to professions, farmers and workers were the top two professions to suffer from IOFB, which might be related to the physical labor involved as well as the poor awareness of safety and lack of protective measures.

Splashing of foreign bodies was the leading cause of IOFB in this research, which is consistent with previous reports [2, 17, 22, 23]. Splashing of foreign bodies appears most commonly during hammering the foreign object; polishing, welding, drilling, and so forth are also common situations [24]. The second and third causes were other explosives and fireworks, which when combined accounted for 31.8%, higher than Zhang et al.'s report (27.8%) [2]. Metal was the first IOFB. The second of the IOFB was nonmagnetic explosives. The difference is probably due to the more explosives used in China's industrial production and the Chinese custom of setting off fireworks.

In this research, we found that for gender differences, male patients were more prone to injuries from splashing of foreign bodies and female patients to stabbing. It is probably because splashing of foreign bodies have to acquire a high momentum to pierce into eyeballs, which are more often available in working environment where males work. In contrast, sharp objects might pierce the eyeball and leave foreign bodies in it, which are viable even with little momentum, such as in cases of scissors or toothpick stabbing. Procedures that give rise to stabbing therefore also involve females. For age and profession differences, we found fireworks accounted for the most in 0–9 year olds and preschool and student patients, while the percentages of patients from splashing of foreign bodies in these populations were lower than those in others. This is inconsistent with Moren et al. who reported that injuries from exercise and toys were common causes of children eye trauma and that injuries from explosives were rare [25]. Reasons might be (1) this research dealt with IOFB only, which accounts for a small percent of eye trauma in children [25, 26]; (2) setting off fireworks is a Chinese folk custom; and (3) there is insufficient guardian supervision over children playing fireworks.

Since IOFB is related to the environment and the protective measures taken, which depend on economic development, incidence of IOFB is lower in developed countries than in developing ones [22, 27]. As China's economy grows rapidly, the living and working environment is also changing quickly. Analyzing the annual variation of IOFB patient population characteristics and the causes will generate more accurate theoretical basis for adjusting IOFB protective measures to the current conditions in China.

In this research, IOFB patients that were mostly males at working ages did not vary significantly over the ten years. As for the variation in number of patients in different age groups, for one thing, patients over 50 were increasing, which might be related to the annually increasing average working age in China [28]. For another, the number of patients between 10 and 19 years old was decreasing over the years, which might be a result of the decreased youth engagement in physical labor. In addition, it is worth noticing that though there were only a small percentage of patients under ten, it did not vary significantly over the years, which suggests that the awareness of safety should be raised in children under ten and their guardians.

For trends in profession distribution of IOFB, on the one hand, the percentages of student and worker patients were declining, which might be attributed to (1) enhanced awareness of safety, (2) less student engagement in physical labor, and (3) improved automation in the Chinese industrial production that creates a safer working environment for workers. On the other hand, the percentage of freelancer patients was climbing, probably because they had no stable long-term job and were disadvantaged in working skills and safety awareness.

In terms of causes, splashing of foreign bodies remained the leading cause of IOFB in the study population over the ten years. Similarly, the percentage of patients from fireworks did not change significantly over the period, which might be explained by the Chinese custom of setting off fireworks and by the unimproved safety awareness in playing fireworks [21]. Apart from that, the percentage of IOFB from other explosives (excluding fireworks) was declining year by year, which might be credited to the improved automation and protective measures in industries with a high risk of explosions, such as industries of coal, metallurgy, and mining.

The incidence of endophthalmitis and retinal detachment in this group was close to the literature [2, 29]. The proportion of IOFB in the posterior segment is higher than that reported by others which is 62%–79% [2, 29]. Vitrectomy is the main surgical method for the treatment of IOFB. Although the majority of IOFB can be removed by means of operation, there are still 2/3 of the patients whose eyes resulted in blindness. Blindness rate is higher in patients with blast injury, occurring endophthalmitis, or with retinal detachment. Blindness rate is lower in 0–9 year-old patients and in patients with foreign bodies in the anterior segment of the eye.

Based on the above analysis, we suggest that males who are over 20 and works as workers, farmers, or freelancers protect themselves against IOFB from splashing of foreign bodies and that females pay attention to protection against IOFB from explosives and stabbing. It is also suggested that students under 20 and preschool children play as less fireworks as possible.

## 6. Conclusion

This research revealed that most IOFB patients were males at working ages and injured by splashing of foreign bodies, which was consistent with other research. However, unlike other research, we also found the difference in causes of IOFB for different populations. Fireworks were the leading cause for patients aged 0–9 while a higher percentage of female patients were injured by stabbing. Moreover, we also found that the percentages of patients over 50 and freelancer patients were increasing while those of patients injured by explosives were declining over the years.

## Figures and Tables

**Table 1:** Distribution of causes by gender.

	Male	Female	Total
	Number/%	Number/%	Number/%
Splashing^∗^	754/59.4%	31/44.3%	785/58.6%
Other explosives	301/23.7%	24/34.3%	325/24.3%
Fireworks	98/7.7%	3/4.3%	101/7.5%
Stabbing	63/5.0%	10/14.3%	73/5.4%
Others	54/4.3%	2/2.9%	56/4.2%
Total	1270/100.0%	70/100.0%	1340/100.0%

^∗^Splashing of foreign bodies.

**Table 2:** Distribution of causes by age group.

	0–9	10–19	20–29	30–39	40–49	50–59	60−	Total
	*N*/%	*N*/%	*N*/%	*N*/%	*N*/%	*N*/%	*N*/%	*N*/%
Splashing^∗^	13/16.9%	80/49.1%	197/67.5%	216/61.7%	192/61.0%	63/57.8%	24/70.6%	785/58.6%
Other explosives	9/11.7%	39/23.9%	49/16.8%	97/27.7%	97/30.8%	29/26.6%	5/14.7%	325/24.3%
Fireworks	32/41.6%	25/15.3%	11/3.8%	11/3.1%	11/3.5%	7/6.4%	4/11.8%	101/7.5%
Stabbing	14/18.2%	14/8.6%	20/6.8%	14/4.0%	7/2.2%	4/3.7%	0/0.0%	73/5.4%
Others	9/11.7%	5/3.1%	15/5.1%	12/3.4%	8/2.5%	6/5.5%	1/2.9%	56/4.2%
Total	77/100.0%	163/100.0%	292/100.0%	350/100.0%	315/100.0%	109/100.0%	34/100.0%	1340/100.0%

^∗^Splashing of foreign bodies.

**Table 3:** Distribution of causes by profession.

	Splashing^∗^	Other explosives	Fireworks	Stabbing	Others	Total
	Number/%	Number/%	Number/%	Number/%	Number/%	Number/%
Farmers	267/62.1%	113/26.3%	19/4.4%	15/3.5%	16/3.7%	430/100.0%
Workers	194/64.9%	88/29.4%	6/2.0%	6/2.0%	5/1.7%	299/100.0%
Freelancers	173/64.6%	56/20.9%	9/3.4%	16/6.0%	14/5.2%	268/100.0%
Employees	82/68.9%	18/15.1%	8/6.7%	4/3.4%	7/5.9%	119/100.0%
Students	38/24.2%	40/25.5%	47/29.9%	22/14.0%	10/6.4%	157/100.0%
Preschoolers	7/25.0%	1/3.6%	10/35.7%	6/21.4%	4/14.3%	28/100.0%
Others	24/61.5%	9/23.1%	2/5.1%	4/10.3%	0/0.0%	39/100.0%
Total	785/58.6%	325/24.3%	101/7.5%	73/5.4%	56/4.2%	1340/100.0%

^∗^Splashing of foreign bodies.

**Table 4:** Distribution of patient age groups by year.

	2004	2005	2006	2007	2008	2009	2010	2011	2012	2013	Total
	*N*/%	*N*/%	*N*/%	*N*/%	*N*/%	*N*/%	*N*/%	*N*/%	*N*/%	*N*/%	*N*/%
0–9	8/6.5%	8/5.6%	4/2.6%	10/5.3%	8/5.0%	11/8.3%	10/7.9%	8/6.6%	5/5.4%	5/5.0%	77/5.7%
10–19	16/13.0%	26/18.1%	30/19.7%	22/11.7%	21/13.2%	12/9.0%	10/7.9%	13/10.7%	9/9.7%	4/4.0%	163/12.2%
20–29	28/22.8%	32/22.2%	31/20.4%	49/26.1%	25/15.7%	28/21.1%	28/22.0%	27/22.3%	23/24.7%	21/21.0%	292/21.8%
30–39	30/24.4%	51/35.4%	38/25.0%	46/24.5%	50/31.4%	37/27.8%	33/26.0%	26/21.5%	18/19.4%	21/21.0%	350/26.1%
40–49	34/27.6%	20/13.9%	41/27.0%	40/21.3%	37/23.3%	36/27.1%	27/21.3%	28/23.1%	23/24.7%	29/29.0%	315/23.5%
50–59	6/4.9%	6/4.2%	7/4.6%	17/9.0%	15/9.4%	5/3.8%	13/10.2%	15/12.4%	13/14.0%	12/12.0%	109/8.1%
60–	1/0.8%	1/0.7%	1/0.7%	4/2.1%	3/1.9%	4/3.0%	6/4.7%	4/3.3%	2/2.2%	8/8.0%	34/2.5%
Total	123/100.0%	144/100.0%	152/100.0%	188/100.0%	159/100.0%	133/100.0%	127/100.0%	121/100.0%	93/100.0%	100/100.0%	1340/100.0%

**Table 5:** Distribution of patient professions by year.

	2004	2005	2006	2007	2008	2009	2010	2011	2012	2013	Total
	*N*/%	*N*/%	*N*/%	*N*/%	*N*/%	*N*/%	*N*/%	*N*/%	*N*/%	*N*/%	*N*/%
Farmers	36/29.3%	48/33.3%	56/36.8%	47/25.0%	48/30.2%	54/40.6%	49/38.6%	41/33.9%	26/28.0%	25/25.0%	430/32.1%
Workers	41/33.3%	46/31.9%	40/26.3%	45/23.9%	43/27.0%	24/18.0%	20/15.7%	11/9.1%	13/14.0%	16/16.0%	299/22.3%
Freelancers	14/11.4%	16/11.1%	19/12.5%	56/29.8%	34/21.4%	20/15.0%	24/18.9%	32/26.4%	20/21.5%	33/33.0%	268/20.0%
Employees	8/6.5%	9/6.3%	6/3.9%	10/5.3%	12/7.5%	12/9.0%	17/13.4%	16/13.2%	15/16.1%	14/14.0%	119/8.9%
Students	16/13.0%	19/13.2%	28/18.4%	20/10.6%	16/10.1%	16/12.0%	12/9.4%	14/11.6%	11/11.8%	5/5.0%	157/11.7%
Preschoolers	3/2.4%	2/1.4%	1/0.7%	4/2.1%	4/2.5%	4/3.0%	4/3.1%	1/0.8%	2/2.2%	3/3.0%	28/2.1%
Others	5/4.1%	4/2.8%	2/1.3%	6/3.2%	2/1.3%	3/2.3%	1/0.8%	6/5.0%	6/6.5%	4/4.0%	39/2.9%
Total	123/100.0%	144/100.0%	152/100.0%	188/100.0%	159/100.0%	133/100.0%	127/100.0%	121/100.0%	93/100.0%	100/100.0%	134/100.0%

**Table 6:** Distribution of causes by year.

	2004	2005	2006	2007	2008	2009	2010	2011	2012	2013	Total
	*N*/%	*N*/%	*N*/%	*N*/%	*N*/%	*N*/%	*N*/%	*N*/%	*N*/%	*N*/%	*N*/%
Splashing^∗^	58/47.2%	93/64.6%	89/58.6%	108/57.4%	92/57.9%	74/55.6%	81/63.8%	72/59.5%	50/53.8%	68/68.0%	785/58.6%
Other explosives	39/31.7%	36/25.0%	42/27.6%	48/25.5%	44/27.7%	30/22.6%	24/18.9%	21/17.4%	20/21.5%	21/21.0%	325/24.3%
Fireworks	17/13.8%	4/2.8%	10/6.6%	5/2.7%	11/6.9%	11/8.3%	18/14.2%	12/9.9%	9/9.7%	4/4.0%	101/7.5%
Others	1/0.8%	5/3.5%	6/3.9%	14/7.4%	4/2.5%	11/8.3%	3/2.4%	4/3.3%	6/6.5%	2/2.0%	56/4.2%
Stabbing	8/6.5%	6/4.2%	5/3.3%	13/6.9%	8/5.0%	7/5.3%	1/0.8%	12/9.9%	8/8.6%	5/5.0%	73/5.4%
Total	123/100.0%	144/100.0%	152/100.0%	188/100.0%	159/100.0%	133/100.0%	127/100.0%	121/100.0%	93/100.0%	100/100.0%	1340/100.0%

^∗^Splashing of foreign bodies.
